# Caveats and Recommendations to Assess the Validity and Reliability of Cycling Power Meters: A Systematic Scoping Review

**DOI:** 10.3390/s22010386

**Published:** 2022-01-05

**Authors:** Anthony Bouillod, Georges Soto-Romero, Frederic Grappe, William Bertucci, Emmanuel Brunet, Johan Cassirame

**Affiliations:** 1 EA4660, C3S Health-Sport Department, Sports University, 25000 Besancon, France; anthony.bouillod@equipegroupamafdj.fr (A.B.); frederic.grappe@equipegroupamafdj.fr (F.G.); 2 French Cycling Federation, 78180 Saint Quentin, France; e.brunet@ffc.fr; 3 LAAS-CNRS, Université de Toulouse, CNRS, 31000 Toulouse, France; gsotorom@laas.fr; 4 Professional Cycling Team FDJ, 77230 Moussy-le-Vieux, France; 5 EA7507, Laboratoire Performance, Santé, Métrologie, Société, 51100 Reims, France; william.bertucci@univ-reims.fr; 6 Mtraining, R&D Division, 25480 Ecole Valentin, France

**Keywords:** metrological properties, mechanical power, gold standard, exercise conditions, statistical analysis

## Abstract

A large number of power meters have become commercially available during the last decades to provide power output (PO) measurement. Some of these power meters were evaluated for validity in the literature. This study aimed to perform a review of the available literature on the validity of cycling power meters. PubMed, SPORTDiscus, and Google Scholar have been explored with PRISMA methodology. A total of 74 studies have been extracted for the reviewing process. Validity is a general quality of the measurement determined by the assessment of different metrological properties: Accuracy, sensitivity, repeatability, reproducibility, and robustness. Accuracy was most often studied from the metrological property (74 studies). Reproducibility was the second most studied (40 studies) property. Finally, repeatability, sensitivity, and robustness were considerably less studied with only 7, 5, and 5 studies, respectively. The SRM power meter is the most used as a gold standard in the studies. Moreover, the number of participants was very different among them, from 0 (when using a calibration rig) to 56 participants. The PO tested was up to 1700 W, whereas the pedalling cadence ranged between 40 and 180 rpm, including submaximal and maximal exercises. Other exercise conditions were tested, such as torque, position, temperature, and vibrations. This review provides some caveats and recommendations when testing the validity of a cycling power meter, including all of the metrological properties (accuracy, sensitivity, repeatability, reproducibility, and robustness) and some exercise conditions (PO range, sprint, pedalling cadence, torque, position, participant, temperature, vibration, and field test).

## 1. Introduction

Power output (PO) [1] measurement during riding is an interesting method to quantify the intensity of exercise produced by cyclists or patients. This measurement is widely used in cycling during training and monitoring [2,3,4,5,6,7] to test or validate mathematical models [8,9,10,11,12,13,14,15,16,17,18], assess the physical potential of cyclists [19,20,21,22,23,24] or measure performance requirements in competitions [25,26,27,28,29,30,31]. In addition, the PO measurement can be used for many research purposes to quantify the effects of rehabilitation programmes [32] or evaluate the fitness level improvement induced by medical treatments, recovery techniques, and many other approaches [33,34,35].

Recently, many systems have become commercially available to provide the PO measurement based on ergometers, home-trainers or mobile systems mounted on personal bikes. Due to the large interest in measuring PO, the list of systems available for this purpose is very exhaustive (including the SRM crankset or pedales, Monark ergometer, PowerTap hub, pedals and chainring, Garmin Vector or Rally pedals, Stages crank arm, Lode Excalibur ergometer, CycleOps-Saris trainer, Cyclus ergometer, Verve Crankset, Shimano Power crankset, 4iiii, Favero pedales, Tacs trainer, Elite Trainer, Rotor INpower cranckset, Look KeoPower Pedales, Quarq crankset, etc.). The validity of power meters is of interest to different potential users. For example, mobile power meters were mainly used by professional cyclists for nearly 25 years. Today, we can observe that all of the WorldTour men’s teams are using power meters, 10 use Shimano power meters, whereas six different power meters (SRM, Power2max, 4iiii, Stages, Quarq, and Rotor) were used by the remaining nine teams. In addition, based on the popularity in the highest level, mobile power meters are actually used by many recreational cyclists. Due to the large number of potential users, manufacturers developed systems and established claims regarding their system’s validity. The validity of power meters has also been tested in scientific research to provide more details regarding their metrological properties (i.e., accuracy, sensitivity, repeatability, reproducibility, and robustness). However, a major problem is that not all of the metrological properties are investigated in most of the studies. Indeed, the studies included only some properties of the power meters and did not provide very relevant information for the end user. Moreover, it is not always easy to make the link between the statistical analyses and the metrological properties studied.

Some misunderstandings are notable from the technological description of systems leading to inappropriate protocols or mistakes during measurement. It is important to clearly understand what is really measured by these systems and the technology used to stress these systems, as well as incorporate all of the recommendations from the manufacturer as calibration and/or offset processes to obtain meaningful data. For example, the SRM system has become the gold standard for power meters to assess the validity of other mobile power meters [36,37,38] or home-trainers [39,40]. Regarding articles focusing on ergometers, various systems are considered to be gold standard systems, such as the calibration rig (Tom, Stanef, SASI, Australia) [41,42], homemade systems [43,44] or metabolic measurement devices [45]. All of these testing processes induce various results that do not result in similar information from each system. In addition, the data analysis of all the studies is not similar regarding periods of measurement, averaging or statistical methods used to compare the results to the gold standard technique. Data analysis and statistical analysis are very important for comparing two systems and directly influence conclusions regarding the validity of the system. Even if the methods of Bland and Altman [46] are perfectly adapted for these issues, many different ways to perform this data analysis have been discussed in the literature.

Previous studies have proposed several conditions to assess the validity of power meters by studying different metrological properties implementing different protocols, data analysis, and testing the responses of the systems according to different exercise conditions (i.e., PO range, sprint, pedalling cadence, torque, position, participant, temperature, vibration or field test) that can directly affect the results and conclusions. No study provides a clear overview regarding the validity of the power meters in all of these conditions. Our own experience over the last decade in power meter testing pointed out that testing situations, methods or environmental conditions, such as temperature or vibration can lead to misinterpretation of confidence that we can get from the devices. Therefore, this review aims to provide caveats and recommendations to assess the validity of cycling power meters, while taking into account all of the metrological properties that should be studied.

## 2. Methods

**Figure 1:**
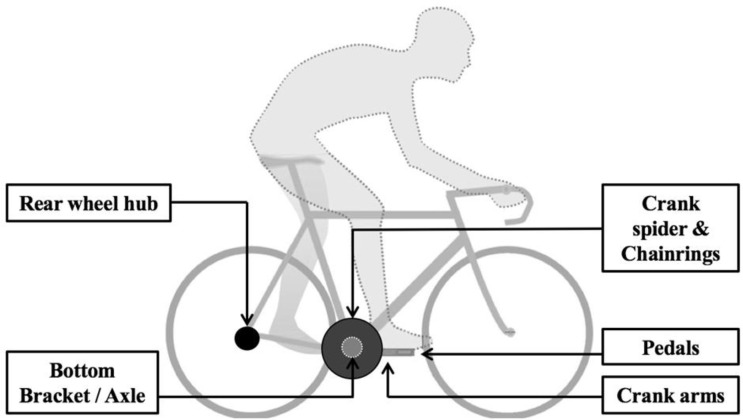
Schematic illustration of power meters’ location on the bicycle.

When performing measurements with power meters, it is legitimate to question the validity of the meters. Validity is commonly defined as the degree to which the devices measure what they are expected to measure [47,48,49]. Applied to cycling, validity is the general quality of the measurement determined by the assessment of different metrological properties, including accuracy, sensitivity, repeatability, reproducibility, and robustness [50]. Accuracy is the ability of power meters to reflect what it is designed to measure [49] or how close the values that are obtained are to the true value. It can be assessed by comparing measurements obtained using a given power meter with measurements obtained using a gold standard power meter. The expected PO and its accuracy can vary according to the location of the power meter on the bicycle. According to the standard instructions of calibration recommended by the manufacturers, the highest PO would be measured at the pedals (e.g., Garmin Vector, PowerTap P1), whereas the lowest PO would be measured at the rear hub (e.g., PowerTap G3, Max One) considering the mechanical losses in the bicycle components (Figure 1) [51]. Frictional losses from the drive train dissipate some of the PO. Indeed, a difference in simultaneous PO measurements should be found before and after the drive train since the data were not compensated during the signal processing. Drive train frictional losses are proportional to the PO and have been suggested to be ~2.4% [8,52].

Sensitivity can be determined as the smallest measurement change that can be detected by the power meter. It is also the ability to detect changes in performance, which may be very small but still meaningful to athletic performance [49]. Sensitivity is a parameter expressing the variation between input and output signals measured by the power meter that can be improved by increasing (1) the number of strain gauges and their location [53] and (2) the sampling frequency. The relationship between both input and output signals is represented by a linear regression most of the time. Moreover, most of the commercially available power meters measure pedalling cadence simply by detecting the complete hub or crank rotations. As a result, when pedalling cadence is low or changes notably within a single rotation (as for a sprint), the power meter’s sensitivity may be affected [2], considering that the pedalling cadence variation is not measured accurately.

Repeatability refers to the variation in repeating measurements with the same power meter under similar conditions [54]. Repeatability implies that the measurements are made under similar conditions with the same equipment, the same place, the same technician, and the same day. In addition, the measurements are made over a short period of time [55], in which the underlying PO can be considered constant. The measurement variations obtained by the same power meter can be ascribed to the measurement process itself.

Reproducibility refers to the variation in measurements made on a power meter under changing conditions [51,54]. The changing conditions may be due to different methodologies (e.g., variations in technicians, equipment, time of day, place, ambient temperature, and innumerable other factors that are not known or cannot easily be controlled) or measurements that are made over a long period of time, within which the PO could undergo a non-negligible change. Reproducibility will be larger than repeatability since it includes components of variance that repeatability does not. A prior estimate of reproducibility is essential when the primary outcome of a study is a quantitative measurement.

Both reproducibility and reliability can also be described in the literature by “reliability”. We can find this terminology in different situations in order to present with-in session reliability as repeatability or with-in device reliability as reproducibility and many other conditions as cadence, ageing, etc.

Finally, robustness is the ability of power meters to remain unaffected by small variations in experimental factors [51]. Therefore, the power meters can be used without failure for a period of time of at least one competitive season (reliability, which can be defined as the consistency of measurements or “the absence of measurement error” [49,56]. In addition, it is the ability to be reliable over time. Many studies assess the validity of cycling power meters at a single moment, usually when the products are new.

In order to identify the articles that are focused on the validity of power meters and to manage this review, we used the Preferred Reporting Items for Systematic reviews and Meta-Analyses (PRISMA) methodology [57,58]. This present review was not pre-registered.

### 2.1. Eligibility Criteria

The main eligibility criteria include all of the studies or conference papers that aim to investigate the metrological proprieties (accuracy, sensitivity, repeatability, reproducibility or robustness) of cycling power meters or ergometers measuring PO for sport, medical or research purposes. Studies were included if (1) they are written in English; (2) they provide a clear methodological content with statistical approaches and gold standard device or method; (3) the full text was obtained to permit effective screening.

### 2.2. Literature Search

A Boolean/phrase search mode was used in the search engine in all of the fields with the following keywords: “Cycling power meter” OR “ergocycle” OR “ergometer” AND “validity” OR “reliability” OR “accuracy” OR “sensitivity” OR “repeatability” OR “reproducibility” OR “robustness” in three different search engines: PubMed, SPORTDiscus, and Google Scholar. The research ended on 10 November 2021.

### 2.3. Studies Filtering and Screening Process

The data extraction process was performed in duplicate on a Microsoft Excel spreadsheet (Microsoft, Redmond, DC, USA) and 4111 items were collected. All of the duplicated references were removed to obtain 2939 items. Based on the criteria described previously, two reviewers (A.B. and J.C.) pre-screened the title and summary in order to obtain a list of the full texts that should be included in the screen list. The screening, which was operated by J.C. and A.B., yielded six additional references from the reference list of screened full texts.

## 3. Results

An important review of the literature listed many studies (74) that were aimed at assessing the validity of PO measurement devices (Table 1). This review provides an exhaustive list of where manufacturers perform PO measurements, including the pedal(s) (e.g., Garmin Vector, PowerTap P1), crank arm(s) (e.g., Stages), spider crank (SRM), chainrings (e.g., PowerTap C1), chain (e.g., Polar S710), bottom bracket axle (e.g., Rotor, InPower), rear hub (e.g., PowerTap G3, MaxOne), home-trainers (e.g., CycleOps, Powerbeam, Wahoo, Elite), and ergometers (e.g., Monark, Lode). All of the systems included their own technology regarding the PO measurement. Moreover, the protocols were very heterogeneous and included different metrological properties, gold standard systems, statistical analyses or exercise conditions. Among the five metrological properties defined in the Methods Section, accuracy was the most studied property (74 studies). Reproducibility was the second-most studied metrological property (43 studies). Finally, repeatability, sensitivity, and robustness were studied considerably less with only 7, 5, and 5 studies, respectively, published in the literature. From our point of view, it is important to investigate the validity of power meters by assessing five essential metrological properties. Unfortunately, we can observe that this recommendation was not followed in the majority of studies. Regarding the gold standard systems, the most common power meter was the SRM. Indeed, the SRM was the reference device in 31 studies. Eleven studies were conducted in the field, whereas all of the remaining studies were performed in a laboratory. Among these 11 studies, five combined both field and laboratory protocols to increase the number of measurements. In addition, the number of participants was very different between all of the studies, from 0 (when using a calibration rig) to 56 participants. In all of the studies that were reviewed, the PO tested was up to 1700 W, whereas the pedalling cadence ranged between 40 and 180 rpm, including the submaximal and maximal exercises. Other exercise conditions have been tested, such as torque, position, temperature or vibrations that will be described later in this article. Finally, the approach for averaging data was also different between the studies, which made the comparisons between the studies difficult. In addition, the 1-s peak PO were often used during the sprint tests.

Among the 74 studies listed, 33 examined the validity of ergometers. The majority of medical bikes measured PO based on the flying wheel resistance, pedalling cadence, and gear. Friction-braked (e.g., Monark), air-braked (e.g., Kingcycle) or electromagnetically braked ergometers (e.g., Lode) can apply theoretical brakes computed from the rotation speed of the flying wheel. The angular velocity of the wheel is different from the pedalling cadence depending on the gear, which is why manufacturers integrate this element into the calculations. For those kinds of bikes, mechanical brakes with a friction belt on the flywheel (e.g., Monark) are very popular, but suffer from many limitations due to the frictional loss as well as the pendulum error of measurement. Ergometers with electromagnetic brakes are probably the best represented category with many medical brands, such as Shiller, Ergoline, Custo, Lode, General Electric, etc. In addition, very few studies report information regarding the validity of these bikes for several reasons. First, it was not easy to install a gold standard system on these ergometers in order to perform simultaneous measurements. Second, these bikes were designed to assess patients and do not fit a cyclist’s requirements for the rider position, saddle, and pedals.

## 4. Discussion

The main findings of this review show that various technology and heterogeneous protocols were reported among the studies, including different metrological properties, gold standard systems, statistical analyses, and exercise conditions. The following sections will discuss the technological description of power meters, protocols, and data analysis, as well as exercise conditions.

### 4.1. Technological Description of Power Meters

Mobile power meters can use different technological components to obtain PO. Regarding force, the systems available on the market mainly use strain gauges, and also resonant string gauges. Regarding angular velocity, manufacturers mainly use magnetic sensors to count the number of passages in front of magnet(s). According to the design of systems, various numbers of magnets are used to obtain a higher sampling frequency on a single revolution (e.g., SRM: 1; Cyclus: 4; PowerTap P1: 20). Finally, several devices measure angular velocity with accelerometers (e.g., Garmin vector, G-Cog). Technological conception is very important since each element will be affected by exercise conditions.

Strain gauges can transform micro-strains into electric resistance variation [122]. Those sensors are pasted on the mechanical part where force is applied. Generally, they are installed in pairs perpendicularly to obtain deformations in two axes. The number of strain gauges on a power meter can be different considering the design of the mechanical part and the accuracy that is expected [53] (e.g., 4–20 strain gauges are used in SRM, depending on the model). From those sensors, an electric signal is filtered and processed to obtain force, while considering the characteristics of the mechanical part. To obtain accurate measurements, several parameters, such as temperature, have to be integrated to adjust calculations. Moreover, calibration (slope adjustment + offset) must be done regularly to adjust the initial deformation of the material over time. The number of strain gauges, the design of the mechanical parts, data processing, and dynamic calculation adjustment play a major role in the accuracy of torque measurements provided by this kind of technology. Alternatively, resonant string gauges can be found in power meters to measure the force at the pedal (e.g., Garmin vectors). This technology is different from strain gauges, even if it provides the same information. Measurements are performed by considering the vibration induced by strain from the mechanical parts. In the same manner as strain gauges, this technology will be affected by signal filtering and processing, as well as temperature.

Regarding angular velocity, the majority of systems measure the time elapsed during a revolution or part of a revolution if several magnets are used. The number of magnets affects the sampling rate of angular velocity and can be a limiting factor during a low rate. In addition, several manufacturers use multi-pole magnets (North–South) to obtain double-point measurements (e.g., PowerTap P1). A higher number of magnets permits the detection of the angular position of sensors to provide a pedalling pattern when computed with the torque. Then, the angular velocity can be determined with an accelerometer (e.g., Garmin Vector and G-Cog). This technology makes it possible to obtain an angular velocity on each point of the revolution. Manufacturers use this advanced technology less due to data filtering. In addition, processing is very important and leads to some limitations. Finally, for a very important pedalling cadence or vibration, systems can be saturated and produce erroneous data.

### 4.2. Protocols and Data Analysis

#### 4.2.1. Simultaneous and Averaging PO Measurement

In this review, 74 studies compare PO measurement systems with gold standard systems to investigate system accuracy (Table 1). Nevertheless, 41 and seven studies investigated reproducibility and repeatability in several consecutive trials. In this situation, the measurement variability cannot be fully attributed to power meters, but it can be attributed to the test and retest variation of participants. This kind of protocol can provide perspectives on using the system, but will not lead to relevant information regarding reproducibility and repeatability.

In addition, some studies compared different ergometers using metabolic measurement devices [45,61,82]. Given that potential PO measurements were obtained with different medical bikes, this kind of protocol could be very helpful for rescaling the training programme according to our goal [123]. Nevertheless, the indirect comparison with another value, such as the oxygen consumption (VO_2_) measurement, could not clearly indicate the level of validity. In those situations, the differences are related to the PO measurement system, participants or metabolic measurements. Using a large sample, overestimation or underestimation can be described if the difference is large enough.

From our point of view, only the simultaneous PO measurement with a system and a gold standard can permit the evaluation of the accuracy of a power meter. In this situation, it is possible to provide quantitative information regarding accuracy. Regarding statistical methods, the Bland and Altman [46,124] method is the most appropriate method to assess accuracy between two measurement systems measuring the same variables. Nevertheless, the simultaneous measurement of PO is not easy regarding synchronization. Studies rarely explain how the synchronization is performed or how data are captured and stored. In this way, it can be imagined that PO data are exported to a spreadsheet with the same sampling rate and are visually synchronized, while maintaining the shape of the data curve. In order to achieve a more reliable signal superposition and avoid human subjective synchronization, we highly encourage the use of the least square method to rescale both signals in the most adequate position. This leads to a reduction at the minimum shift between both signals, as proposed by Hermand et al. [125] for a comparison of heart rate signal and as recently promoted by a recommendation for the evaluation of the heart rate measurement system [126].

**Figure 2:**
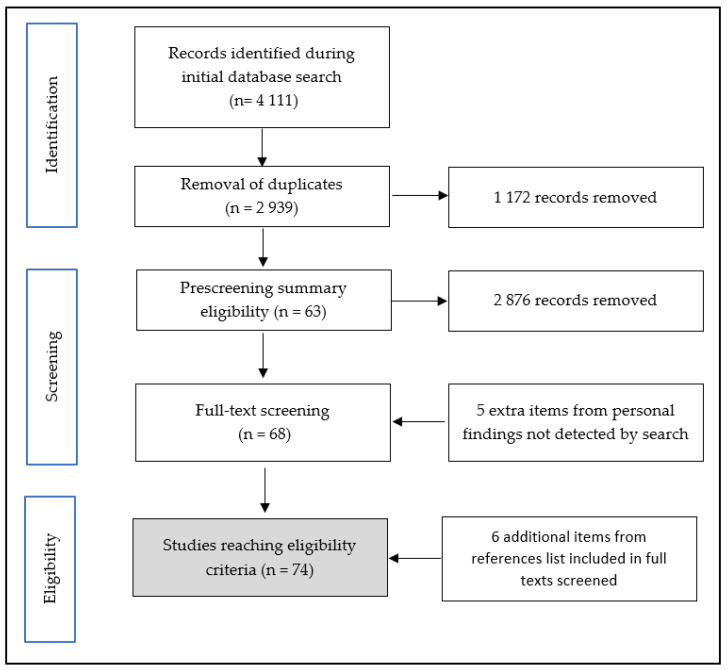
Flowchart of inclusion and screening process for studies’ eligibility.

Moreover, instantaneous PO values are cyclic due to the different force moments and effectiveness during the pedalling cycle [127]. Even during the constant PO measurement, instantaneous PO follows a “sinusoid trend” around an average value (Figure 2). Therefore, synchronization is very important in the comparison of small timing periods. Many users believe that pressing the record button simultaneously would be sufficient to obtain an acceptable synchronization. In fact, this part does not totally fix the synchronization problem. Bike computers are only a passive recorder of sensor data and capture data when they are available. This indicates that the PO measurement systems must be started simultaneously without any button or possibility of triggering those systems. The PO measurement system started sending PO data after the initialization process (unknown time) and movement detection. For example, the SRM system starts the initialization process and then the measurement follows after the crank rotation is detected by a reed switch.

**Figure 3:**
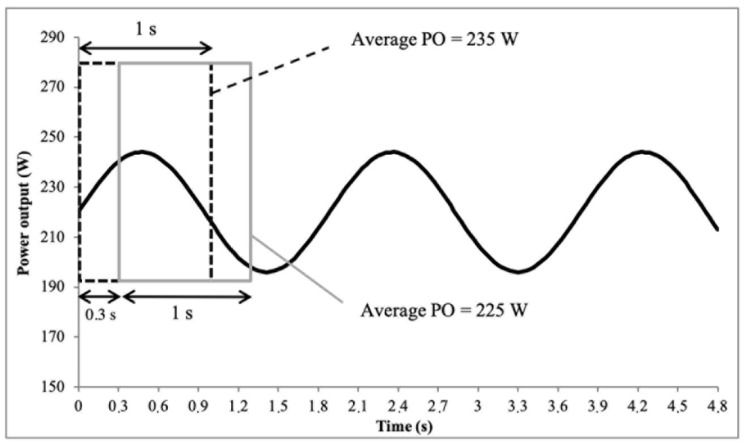
Representation of power output (PO) values with two different periods of 1 s shifted by 0.3 s.

These elements led to an inability to perfectly synchronize many systems and placed important limitations on a statistical approach when analyzing the 1-s peak PO. PO values follow a sinusoidal curve. A shift in synchronization automatically leads to the average PO for different periods and finally produces different average values. For example, Figure 3 highlights a PO value of approximately 220 W with two different average time periods of 1 s. Even if the PO curve is the same, both 1-s periods had an average difference of 10 W. This indicates a caveat in PO measurement comparison and leads to a statistical analysis that had very large limits of agreement (LoA) and poorer accuracy than expected.

**Figure 4:**
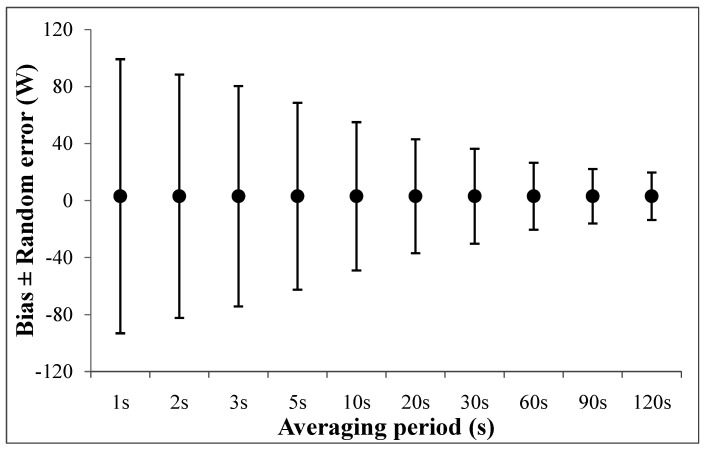
Bias ± random error of Stages power meter compared to SRM power meter during the 3 h field training session (personal data). Each point corresponds to a specific averaging period in order to analyze the same data samples.

At the same time, many studies compared the PO measurement with average values delivered by two sensors. The duration of the averaging period can be very different from one study to another. The period length ranged from 1 s, 5 s [40], 10 s [85], 15 s [37], 30 s [71], and 1 min [99] up to several minutes [102,103]. This very large difference in data processing will directly modify the LoA highlighted by the Bland and Altman [124] analysis. A comparison between Stages and SRM power meters has been made to illustrate the effect of averaging periods on the LoA (Figure 4). Based on the same samples, data processing was performed using averaging periods of 1, 2, 3, 5, 10, 20, 30, 60, 90 and 120 s. A short averaging period provided a larger LoA compared to the longer periods. Therefore, the 1-s duration cannot be used to provide a comparison during the measurement due to the uncontrollable synchronization process. Conversely, periods over 30 s reduce the difference and lead to a smaller LoA. Given that instantaneous values measured by systems can be lower or higher than real measurements, the average of both negative and positive differences cancels the observed discrepancy. This process does not modify bias, but increases the random error.

To standardize studies regarding the PO measurement, the averaging period seems to be a very important method that permits the comparison of power meters. A small averaged period can be interesting to compare systems and obtain information regarding the sensitivity of PO variations. Nevertheless, a systematic bias due to a synchronization shift should be avoided when comparing data. In this context, a 5-s duration appeared to be a relevant period for sub-maximal intensities since users often use smoothing to display the PO data on a bike computer (the average was 3 or 5 s). This duration provides less variation in the power control display that allows for the target PO to be maintained more easily during the effort.

Averaging periods longer than 5 s lead to a smaller LoA (Figure 4). This approach will ensure the validity for each point of the record power profile (RPP) [24] except for a 1-s period. Indeed, it is not possible to use this period to compare measurements since it enlarges LoA. However, the explosive capability of an athlete or patient is a very important parameter to consider [128,129]. To investigate that characteristic (maximal PO or time to peak PO), it will be very important to specifically investigate it through different protocols [130,131]. The first recommendation is to perform a test to reduce the random error related to the time shift [132]. This can be achieved by increasing the number of participants and performing several trials for each participant (ranging from 5 to 10 s). Second, to obtain the maximal PO from participants, it is very important to use the PO measurement system with an accurate measurement in a high value with higher sampling rates from a gold standard system and power control. For example, an SRM system provides PO at 4 Hz, but only the bike power control provided by the SRM Company (power control 7 and 8) records those values. If the recording was made by another brand (Garmin, Bryton, Suunto), the recording is conducted only at 1 Hz. These two recommendations reduce error in data processing and facilitate the comparison of maximal PO values with gold standard systems.

#### 4.2.2. Statistical Methods for Assessing the Validity of Power Meters

All of the metrological properties of the power meters have been analyzed using many statistical methods in the literature. It should be noted that the studies reviewed in Table 1 did not always describe the statistical methods in detail, according to metrological properties.

Different methods are used in validity studies of power meters (Table 2). The circumstances in which these methods can be applied vary, and some are never appropriate for assessing validity. All of the methods are reviewed briefly regarding their use for evaluating power meters.

The paired *t*-test would be used to compare the mean values between two different power meters (i.e., accuracy, sensitivity, and robustness) or between test and retest sessions (repeatability and reproducibility) to analyze whether there is any statistically significant bias between the power meters or tests. Although the paired *t*-test is useful, it should not be employed on its own as an assessment of all the metrological properties. Bland and Altman [133] stressed caution in the interpretation of a paired *t*-test for assessing accuracy, since the detection of a significant difference is actually dependent on the amount of random variation. For conducting more measurement sessions, the analysis of variance (ANOVA) is needed. ANOVA with repeated measures has been used to assess systematic bias between several measurements with appropriate post hoc comparisons. However, the limit of this statistical analysis is similar to the limits of the paired *t*-test. The systematic bias is affected by a large random variation.

Altman and Bland [134] introduced a method to assess accuracy between two methods. For each pair of values, this method uses the difference between two measurements along with the mean. The mean of the difference estimates the bias, which is a tendency for one of the methods to provide consistently higher or lower values than the reference method. Then, the LoA analysis presents and explores the data of the two methods with a Bland-Altman plot, which represents the differences between means [46]. It is important to observe whether there is any heteroscedasticity in the data. If the heteroscedasticity correlation is close to zero and the differences are normally distributed, a researcher could calculate the limits of agreement as follows: Mean of the two methods ±1.96 × SD of the differences between the two methods. The ninety-five percent confidence interval (95% CI) is formed by low and high limits of agreement. If heteroscedasticity is suspected, Bland and Altman [124] recommend the logarithmic transformation of the data before the calculation of the LoA. The final step would be to antilog the data. Regarding the interpretation of the LoA, it is a scientist’s task to judge whether the LoA are narrow enough for the test to be of practical use. The greater the random error, the larger the minimal detectable change would be for a given power meter.

The correlation coefficient is used by some authors for assessing the accuracy between two power meters [36,39,60,66,67,70]. A power meter is sufficiently accurate when a high (>0.9) statistically significant correlation coefficient and linearity are obtained [135]. However, Bland and Altman [133] and Sale and Norman [136] considered the use of the correlation coefficient to be inappropriate, since it cannot assess systematic bias. The linear regression analysis is another useful method for analyzing the agreement between two methods and enables a visual inspection of the strength of agreement. Similar to hypothesis tests for bias and correlation methods, regression analysis may be misleading in some accuracy assessments [133,137]. Sample heterogeneity is, again, a possible problem for extrapolation of the statistical analysis. For systematic bias, the null hypothesis that the intercept of the regression line equals zero would be tested. As for the paired *t*-test, a wide range of individual differences may lead to false acceptance of this hypothesis.

The coefficient of variation (CV) is also used to determine if the measurements are repeatable and reproducible [135]. It is important to take into account the magnitude of the smallest worthwhile change and the uncertainty or noise in the test results. The detectable change in performance represents a magnitude of less than 2% in elite athletes. Indeed, in sport sciences, Paton and Hopkins [138] reported a variation in performance from ~0.5% in road races up to ~2.5% in mountain biking events. In a recent study, the mean CVs obtained with the PowerTap, Stages and Garmin Vector devices are slightly higher than 2%, but the statistical analysis indicates that the four power meters provide reproducible PO during submaximal tests in the laboratory [51]. Van Praagh et al. [139] proposed a 5% margin of error to consider power meters as repeatable or reproducible, but this margin is too wide to detect a small change in performance. When considering an elite athlete with a maximal aerobic power of 400 W, that margin of error represents 20 W. Assuming that the accuracy claim of the SRM is correct, the results indicate that the PowerTap, Stages and Garmin Vector power meters have an accuracy of ± 2–3% for PO between 150 and 350 W. In other studies, the CVs were similar for the PowerTap hub system (CV = 2.1%), Polar S710 (CV = 2.2%), and the Ergomo Pro (CV = 4.1%) [36,77,103]. Hurst, Atkins, Sinclair, and Metcalfe [88] reported higher CVs for both the Stages (CV = 5.5%) and SRM (CV = 5.1%) power meters, but this result was biased by trail vibrations and a small variation in pacing in the different trials. Higher CVs were also reported for the G-Cog BMX [67,70] and the Look Keo Power [38] power meters.

Intraclass correlation coefficient (ICC) is also a common method for assessing repeatability and reproducibility [38,41,60,71,109,140]. It is based on the terms used in the calculation of the F-value from repeated measures ANOVA. The main advantages of this statistical analysis is that the ICC is univariate and it can be used when more than one retest (or power meter) is compared with a test (or gold standard) [56]. The ICC can be calculated in a way that is sensitive to the presence of systematic bias in the data. A more detailed approach would be to calculate confidence intervals for a given ICC, as shown by Morrow and Jackson [141]. The ICC includes variance for individuals and is therefore affected by sample heterogeneity to a degree that a high correlation may still lead to an unacceptable measurement error for some analytical goals [142,143].

Finally, in studies that compare different power meters to assess accuracy, the effect size (ES) represents the magnitude of the difference between power meters. The absolute ES is the difference between the average or mean outcomes between the two different power meters [144]. While the P-value can inform the reader whether an effect exists, the P-value will not report the size of the effect. Both of the substantive significance (ES) and statistical significance (P-value) are essential results that should be reported. ES can refer to the raw difference between a power meter’s means, as well as standardized measures of exercise conditions. ES can also quantitatively compare results from different studies and thus can be used in meta-analyses. The most commonly used ES is Cohen’s *d*, which represents the ratio of the mean difference over the pooled variance to estimate the magnitude of the difference. As Cohen [145] proposed, the difference was considered trivial when ES ≤ 0.2, small when ES ≤ 0.5, moderate when ES ≤ 0.8, and large when ES > 0.8.

### 4.3. Exercise Conditions

Due to technological differences, integrated components or measurement location, many exercise conditions can alter the PO measurement, including PO range, sprint, pedalling cadence, torque, position, participant, temperature, vibration or field test. The following paragraphs aim to describe some of the problems that occur with those exercise conditions and explain why it is necessary to assess them.

#### 4.3.1. Power Output Range

The range measurement is the area determined by minimal or maximal values of the variable to be measured (PO, in this case). For example, the SRM power meter has a range measurement between 10 and 2500 W, which allows the valid measurement of PO during low-intensity (e.g., rehabilitation protocols) and high-intensity (e.g., sprint ability in world class track or BMX cyclists) exercises. This paragraph does not take care of the maximum PO during sprints, since those cases will be managed separately in another paragraph.

Testing many PO ranges is necessary to confirm that a system can provide valid data in a PO range, in which the system is supposed to be used according to the population and need. As an example of rehabilitation, tools or medical devices should be tested from 0 to 300 W and systems aiming to test elite athletes should be tested from 0 to 600 W during a steady state of at least 1 min. Higher values are not supposed to arise during the steady state measurement and will be managed in the sprint section. Many studies aimed to investigate PO systems using an incremental protocol to obtain comparisons of different PO values [42,51,77]. When a study is performed with a calibration rig system or other systems driven by an engine, it is very easy to perform a large PO range over 450–500 W. In other cases, it could be difficult to find participants that are able to maintain this intensity for least 2 min. It is only possible to perform different steps at a high intensity with a recovery phase.

**Figure 5:**
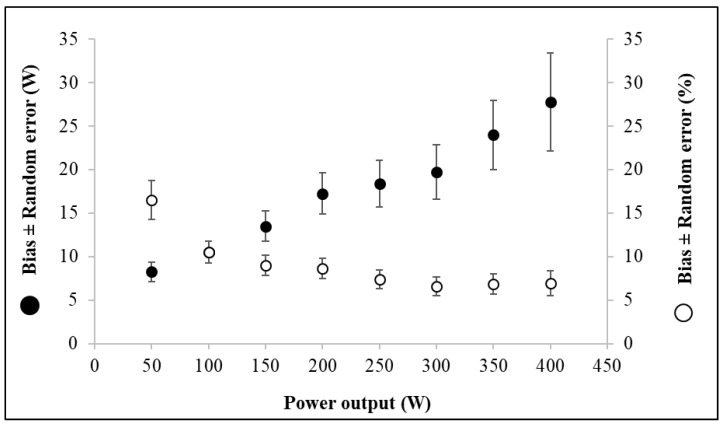
Comparison between two power meters (personal data). The graph represents the absolute bias ± random error expressed in Watt (black markers) and the relative bias ± random error expressed in % (white markers) for each power output (PO) level tested.

Several studies reported problems in the linearity of measured PO with drifts in validity and larger LoA in higher values [51,63,77,86,140]. Nevertheless, some studies did not provide a very clear overview of validity functions to a PO range. When the Bland-Altman analysis is performed for all of the PO data, the shape of the plot provides an idea of the trend, but not relevant data to confirm the agreement. To provide relevant information, each PO range should be analyzed separately with the methods of Bland and Altman, after log transforming the data where heteroscedasticity is observed, in order to provide the bias ± random error for each range (Figure 5). This information permitted us to clearly observe if the PO system had a different bias with a higher value as an example that indicates drift in the measurement process. In other cases, this method highlights a larger interval between LoA that indicates the modification of sensibility function throughout the PO range.

#### 4.3.2. Sprint

Sprint tests are widely used in cycling to assess the anaerobic capacities of athletes [146,147], and the Wingate test [148,149] and force-velocity test are the most popular approaches [150,151,152]. Those kinds of evaluations are characterized by a brief all-out period of cycling (from 5 to 30 s), in which maximal values are carefully collected. These tests provide 1-s peak PO, time to peak PO, and some other parameters, such as the 5-s average peak PO and PO decrease [146]. During those very intense PO phases, the power meter has to be very reactive to perform measurements accurately and be able to measure very high values (up to 2000 W for elite track riders). In the past, several studies noted the limitations of the friction belt system to assess PO during sprints due to the inertia of the flying wheel [95,99,153,154]. More recently, Rodger, Plews, McQuillan, and Driller [113] reported an unacceptable level of agreement during high PO (>650 W) with Cyclus and Stages power meters. Bertucci, Duc, Villerius, Pernin, and Grappe [36] also reported a decrease in accuracy during sprints with PowerTap hub SL+. Considering the measurement conditions, several authors noted that the sampling rate of measurement had a major role in accuracy for maximal PO during sprints [132,155]. A higher sampling rate allows for better accuracy in 1-s peak PO determination, but no difference was found for the 5-s average peak PO. For this reason, it is very important to perform a comparison during sprints with a gold standard system at the best sampling rate as possible. Sprints or intermittent exercises with an accurate measurement of PO are performance issues, since these exercises can be integrated into therapy for pathologies, such as obesity [156], artery disease [157] or for cardio-respiratory benefits [158]. Sprint assessment is a specific evaluation in which the technical requirements from the equipment must be adequate for this purpose. If a system aims to perform measurements at a high level of PO (over 600 W) or during explosive exercise bouts, it is highly recommended to assess the system in those situations to verify their capabilities.

#### 4.3.3. Pedalling Cadence

PO is commonly obtained by measuring both angular velocity and torque. For each PO range, an athlete or patient can vary these two parameters to adjust their PO with cadence or resistance. Pedalling cadence during a constant PO exercise bout can be very different for rehabilitation purposes (15–100) [159,160] or performance cycling (70–120) [161,162]. Due to technical differences in angular velocity measurement solutions (single magnet, multi-magnets or accelerometer(s)), several studies reported alterations in accuracy after changing the pedalling cadence. Bouillod, Pinot, Soto-Romero, Bertucci, and Grappe [51] or Duc, Villerius, Bertucci, and Grappe [77] reported alterations of PO measurement using different cadences. If the PO measurement is performed on pedals, the crank arm or bottom bracket axle, the pedalling cadence will be directly integrated into the PO calculation. In addition, if the PO measurement is performed on the rear wheel (hub or axle), the angular velocity of the wheel is integrated into the calculation. Technically, most of the commercially available power meters measure the angular velocity simply by detecting the complete hub or crank revolution with one or several magnet systems. In those cases, system measurements on the rear wheel obtain a higher number of revolutions for one single pedalling revolution according to the gear ratio. Those systems have the advantage of measurements that are made with a very low pedalling cadence and that obtain more angular velocity values. Some systems can also use several magnets on the wheel (Monark 894 E, Cyclus 2) or inside the pedals (PowerTap P1) to obtain more data and improve the accuracy of the pedalling cadence measurement. In addition, some systems measure angular velocity based on accelerometers (e.g., Garmin Vector and G-Cog). It has been established that accelerometers can be less accurate with a very low speed movement and can saturate at a very high velocity [163]. Given the relationship between PO, cadence, and torque, it is not certain that PO measurement modifications are due to changes in cadence or are directly affected by angular velocity or torque measurements. For the same PO range, an increase in pedalling cadence will lead to a decrease in torque and vice versa.

#### 4.3.4. Torque

Torque measurement is one part of the calculation. Unlike pedalling cadence, torque is a little more difficult to manage for the same PO range, given that it depends on the sensor sensitivity and location. Nevertheless, several authors created protocols that induce different ranges of torque. Bertucci, Duc, Villerius, Pernin, and Grappe [36] created a protocol on a treadmill with various gear ratios, slopes, and speeds to test the influence of torque on the PowerTap hub’s accuracy. Furthermore, Bernard, Decatoire, and Lacouture [65] used an engine system to produce various torque values on an SRM power meter and investigate the impact of torque variations on accuracy. Regarding torque testing, tests of several PO ranges with different pedalling cadences, gears or slopes could be sufficient to assess various torque ranges.

#### 4.3.5. Position

The effects of position on PO measurements have been tested in several studies [36,51,69,77,89,94,106] to assess the sensitivity of power meters. Bertucci, Duc, Villerius, Pernin, and Grappe [36] reported that the PowerTap rear hub was not significantly affected by the position change (standing vs. seated) when compared to the SRM power meter. Duc, Villerius, Bertucci, and Grappe [77] observed a significant decrease in PO measured in the standing vs. seated position by the Ergomo pro at 15 km/h and 6% on a treadmill, while the PO measured by the SRM was unchanged. Finally, Bouillod et al. [51,69] also showed an effect of cycling position on the PO measured by SRM, PowerTap G3, and Stages. For the same velocity on a treadmill, the PO was higher in the standing position for both the SRM (+2.1%) and the PowerTap G3 (+2.4%) power meters. This increase in PO is due to the mechanical deformation in both of the tires and the bike frame. Indeed, the increase in peak pedal force during the downstroke phase [164] occurs according to Newton’s second law (F = m × a) and induces an uneven work rate generation that enhances the acceleration and deceleration periods during the crank cycle. The force variations during the overall cycle could explain the mechanical losses measured in the standing position. In contrast, the PO measured by Stages was lower in the standing position (−4.4%), whereas the Garmin Vector pedals were not affected by the change in position. The authors hypothesized that the PO measured by Stages was significantly lower in the standing position, which probably occurred due to the left-crank-only measurement. Indeed, the algorithm used to determine PO for the Stages system simply doubles the value determined at the left crank and then creates an average. This process may create some issues in situations for which a contralateral force production imbalance is present. The Garmin Vector system did not measure the PO change between the seated and standing positions. This could be due to some technological limits of the system that will be discussed further below. To conclude, both SRM and PowerTap rear hub power meters are considered sensitive for measuring the PO change between the seated and standing positions, whereas the Ergomo pro, Stages, and Garmin Vector power meters are not.

#### 4.3.6. Participant

The number of participants and their characteristics are very important for integrating the results that were obtained. Some studies used only one participant during the measurement process [36,51,77,88,89]. In this situation, the authors consider that differences in the pedalling technique between the athletes or patients does not affect the accuracy of measurement. However, some studies reported differences in the pedalling technique between the novice and elite athletes [165] or between cycling specialities [166,167]. These inter-individual differences can be observed with power meters using the torque analysis. For example, a recreational cyclist or patient has higher torque amplitude values during pedalling for the same PO. Technically, it cannot be excluded that a smoother pedalling pattern can be measured more accurately than an erratic pattern. Given the sampling rate measurement of both force and cadence, it could be possible that some data are interpolated and generate more error with an erratic pedalling pattern. In addition, many systems measure PO in the left and right sides separately (pedals and crank arms). Several studies measured some differences in the pedalling technique for the pulling/pushing technique [168,169,170]. To confirm that the PO measurement system can be sensitive and accurate for all those pedalling techniques, testing many participants during comparisons appears to be mandatory. The pedalling technique could be measured using effectiveness and smoothness indexes. Effectiveness measures how much of the PO that is delivered to the pedal is pushing it forward, whereas smoothness measures how smoothly the PO is delivered to the pedal throughout the revolution. More recently, several systems appeared on the market to measure the PO with only one side device and doubling values to obtain the entire PO. During comparisons with these sensors, the accuracy that was obtained is directly impacted by the bilateral symmetry of the participants that were tested and cannot be imputed fully to the sensors. Many studies reported that the pedalling symmetry is related to many factors, such as pedalling cadence [171], workload [172,173,174] or fatigue [174,175] and that the symmetry can be reduced using specific training approaches [176]. For those kinds of sensors, it is recommended that several participants should be tested during the comparison. Nevertheless, the results that are obtained cannot guarantee that the accuracy of PO will remain constant in various situations, since the symmetry of riders can be modified and lead to higher error measurements.

#### 4.3.7. Temperature

The use of strain gauges allows for the accurate measurement of torque, but they are sensitive to changes in ambient temperature [42,177], which affects the calculated PO. Indeed, an increase in ambient temperature induces a change in the offset, which leads to a systematic error in the measured PO. As reported by Wooles, Robinson, and Keen [177], the offset changes an average of 7 Hz per degree for the SRM, which represents approximately 2 W. Two other studies were interested in analyzing the effects of temperature on PO measurements. Davison, Corbett, and Ansley [75] showed that the CompuTrainer significantly underestimated the SRM by ~7 W in low temperatures (15 and 20 °C), but was similar under high temperatures (28 and 35 °C). In addition, Gardner, Stephens, Martin, Lawton, Lee, and Jenkins [42] stated that both the SRM and PowerTap power meters are sensitive to differences in temperature. More recently, Shute et al. [178] compared four systems (CompuTrainer, PowerTap Hub, Stages crank, and Garmin vector) in three different environmental conditions of temperature, cold 7°, regular 20°, and hot 33°. In this study, the authors observed a significant alteration of accuracy for PowerTap, Vector, and Stages, especially in the hot situation. These studies show that care is needed when performing an offset, especially at the start of the ride if the bicycle is moved from a warm to a cold location. It is recommended to set the zero offset (when possible) on power meters after a warm-up period in order to ensure that the components (primarily strain gauges) are “acclimated” to the ambient temperature. Otherwise, it is recommended that a system that compensates for temperature variations is used during the ride.

#### 4.3.8. Vibration

The effect of vibration on PO measurements has been tested in only three studies [51,114,115]. Bouillod et al. [51] used vibrating plates (Globus, Physioplate FIT, Italy) and demonstrated that the PO measured by Stages and Garmin Vector was significantly decreased by high vibrations (48 and 52 Hz, respectively), whereas the PO measured by SRM and PowerTap G3 power meters were not. Rodríguez-Rielves et al. [114] observed an alteration of Favero Duo measurement when performed with the vibration set at 30 and 40 Hz, which is generated by the Merit Fitness V2000 vibration plate. In the second study, Rodríguez-Rielves et al. [115] pointed that the Rotor InPower system seems to not be influenced by the similar vibration process.

These results can suggest that the robustness of the Stages and Garmin Vector and Favero Duo could alter the validity of the PO measurement on roads with cobblestones or with high macrotexture surfaces. In addition, the decrease in PO measured by Stages and Garmin Vector and Favero Duo could be due to the use of accelerometers to measure angular velocity. The accelerometers could be out of their range of measurement, especially when the road conditions involve a severe vibration exposure from 30 Hz or 48 Hz. The Stages and Garmin Vector power meters cannot be considered suitable for practice with strong vibrations, such as mountain biking and cobblestone roads. The study of Bouillod, Pinot, Soto-Romero, Bertucci, and Grappe [51] highlighted the importance of testing the robustness of the power meters with vibrations, considering that the PO measurement could be affected by the technological conception of the systems.

#### 4.3.9. Field Test

All of the studies analyzed the PO systems in several steady-state periods. During those steady-state periods, small variations in PO are observed around target values. This situation can permit a PO range to be associated with a specific accuracy. Nevertheless, this protocol offers a situation with very low PO variation and without free wheel periods, as in natural-use scenarios. A recent study [21] showed that the variations in PO were lower with ergometer conditions (CV = 6.8%) compared to the level ground (CV = 14.5%) and uphill (CV = 14.1%) conditions for similar exercises. These results suggest that the PO fluctuations were significantly higher under road-cycling conditions by considering the different techniques, vibrations, and pacing strategies. In cases of sensors that lack sensitivity, the PO could be impacted substantially by variations. To recreate real situations, many authors simulated time trials [36,109,140]. In that kind of situation, if a simulation included positive and negative slopes, it is possible to recreate PO measurements that closely simulated the real use. In addition, if the comparison focuses on mobile PO measurement systems, it is highly recommended to perform comparisons in field tests to include many natural stresses that were described previously, such as temperature variation, a large range of PO, and cadence. Few studies tested mobile PO systems in the field [36,51,67,69,70,77,88,89,100,102,103,104]. Laboratory tests were used to test each one separately in order to check for potential problems or a lack of sensitivity under specific conditions and provide explanations. This protocol leads to smooth external stresses and provides steady-state situations that are not in phase with the final use.

## 5. Conclusions

The procedures and methods used in the studies to assess the validity of power meters are very different, including the protocols, statistical analysis, data processing or interpretation. These differences are mainly due to a lack of consensus in the literature to evaluate these tools. As described in this review, the methods used to assess power meters largely affect the results and make the comparisons between the studies and devices impossible. This review provides some caveats and recommendations to assess the validity of cycling power meters, such as including all of the metrological properties (i.e., accuracy, sensitivity, repeatability, reproducibility, and robustness). In addition, several exercise conditions that could affect the validity have been noted. All of these metrological properties and exercise conditions have to be tested to assess the validity in any situation. However, given the large number of these metrological properties and exercise conditions, the studies can be performed with only some properties, if not all of them are required (e.g., vibration for ergometers). If some properties are not tested, they should be mentioned in the Study Limitation Section. To provide relevant information to the readers, it is recommended to compare a device with a gold standard system using similar time measurements. In addition, the PO range has to fit the requirements of a population that use these systems (rehabilitation bike, elite athletes). Moreover, the inclusion of a large number of participants (at least 10) is highly recommended, in order to limit the effects of individual characteristics on validity. Finally, the protocols aimed at evaluating sensors during anaerobic tests (or sprints) should be performed to obtain specific data, similar to the maximum PO or times to reach a maximum PO. Furthermore, the performance of at least three trials for each sensor and the evaluation of the reproducibility or repeatability between the sensors are also recommended.

## Figures and Tables

**Table 1:** Overview of the studies included in this review.

Study	Power Meters	Gold Standard	Lab	Field	n	Power (W)	Cadence (rpm)	Data Averaging	Metrological Properties	Exercise Conditions
Abbiss et al. [59]	Velotron Ergometer (Racer-Mate, Inc., Seattle, WA, USA)	Calibration rig	x		15	250–1700	100–120	Max and full step	Accuracy Reproducibility	Sprint—Time trial 30 km
	SRM Scientific	Calibration rig	x		15	250–1700	100–120	Max and full step		Sprint—Power range—Time trial 30 km
Astorino and Cottrell [60]	Velotron Ergometer	Monark 894E (Monark, Vansbro, Sweden)	x		40	0–11 W·kg^−1^	140–180	Average on Wingate (30 s) and peak value	Accuracy Reproducibility	Sprint (Wingate)
Attaway et al. [61]	Monark Ergometer and Velodyne Ergometer (Frontline Technology, Inc., Irvine, CA, USA)	Physiologic and perceptual responses	x		7	100–400	90 with Monark and Free with Velodyne	Full step	Accuracy Reproducibility	Power range
					6	125–225	90	Full step		Power range
Balmer et al. [62]	Kingcycle Air-Braked Cycle	SRM	x		9	400	-	60 s	Accuracy Reproducibility	-
Balmer et al. [63]	Kingcycle Air-Braked Cycle Ergometer	SRM	x		13	360–500	-	60 s	Accuracy	MAP-16.1 km TT
Balmer et al. [64]	Monark 814E	SRM Scientific	x		56	300–500	-	Average on Wingate (30 s) and peak values (1 and 5 s)	Accuracy	Sprint (Wingate)
Bernard et al. [65]	I-Crankset (SENSIX Society, Poitiers, France) and SRM	RTSL (Eaton Corporation, Troy Michigan, USA)	x		1	Unknown	56–90	Full step	Accuracy	Torque range—Cadence range
Bertucci et al. [36]	PowerTap PRO + (Saris Cycling Group, Madison, WI, USA)	SRM Scientific	x	x	1	100–420 + sprint	45–120	Full step	Accuracy Sensitivity Reproducibility	Power range—Sprint-Position (Seated vs. Standing)—cadence
Bertucci et al. [39]	Axiom Powertain (Elite, s.r.l., Fontaniva, Italy)	SRM Scientific	x		19	130–400	90 during MAP test ~90–100 during TT tests	Full step	Accuracy Reproducibility	Power range—Slope
Bertucci et al. [44]	New ergometer	SRM Scientific	x		1	100–300	90	Full step	Accuracy Reproducibility	Power range
Bertucci [66]	Fortius cycling ergometer (Tacx, Wassenar, Teh Netherlands)	PowerTap	x		35	200–350	Unknown	Full step	Accuracy Reproducibility	Time trial 6 and 30 min
Bertucci et al. [67]	G-Cog (Rennen Design Group, USA)	SRM and PowerTap Pro	x	x	1	100–1050	-	Sprint 30 s	Accuracy Reproducibility	Sprint-Power range
Bini and Hume [68]	I-crankset system (Sensix, France)	Lode Excalibur	x		17	1.5 to 3.5 Watts/Kg	60–80–100	10 crank cycles	Accuracy Reproducibility	Power range
Bouillod et al. [69]	Stages one-sided (Stages Cycling, Saddleback Ltd., UK) and Garmin Vector (Olathe, KS, USA)	SRM Professional	x	x	1	150–1400	60–100	Peak 1 and 5 s during sprints and full step during sub-maximal incremental tests, sub-maximal 30-min continuous tests and field test	Accuracy Sensitivity Reproducibility	Power range—Cadence range—Sprint—Field—Position—Time
Bouillod et al. [51]	Stages one-sided, Garmin Vector and PowerTap G3	SRM Professional	x	x	1	150–1600	60–100	Peak 1 and 5 s during sprints and full step during sub-maximal incremental tests, sub-maximal 30-min continuous tests, field test and vibration test	Accuracy Sensitivity Reproducibility Robustness	Power range—Cadence range—Sprint—Field—Position—Time—Vibration
Chiementin et al. [70]	G-Cog (Rennen Design Group, USA)	Uniaxial accelerometer (DJB A/120/V sensitivity: 100 mV/g)	x		6	100–400	Unknown	Full step	Accuracy Reproducibility	Power range
Costa et al. [71]	PowerCal (CycleOps, Madison, WI, USA)	Velotron ergometer	x		10	400–700	Unknown	Peak and mean 15, 30 and 45 s	Accuracy Reproducibility	Power range
Costa et al. [72]	PowerCal (CycleOps, Madison, WI, USA)	Velotron ergometer	x		21	~280	Unknown	Intervals of 1 km averaged	Accuracy Reproducibility	Time
Costa et al. [73]	Stages one-sided	Velotron Ergometer	x		26	100–350	90–115	Full step	Accuracy Reproducibility (two trials)	Power range Time
Czajkowski et al. [74]	PowerTap P1 (Saris Cycling Group, Madison, WI, USA)	SRM Professional	x		5	150–1250	60–100	Peak 1 and 5 s during sprints and full step during incremental	Accuracy Reproducibility	Participant—Power range—Cadence range-Sprint
Davison et al. [75]	Computrainer ergometer (Racermate Inc., Seattle, WA, USA)	SRM	x		1	200	~90	Full step (2 min)	Accuracy Robustness	Temperature—Calibration procedure
Dickinson and Wright [76]	Garmin vector 3	Lode Excalibur Sport			7	100–350 W + Sprints	Free	Full step (2 min) Peak power 10 s–on sprint	Accuracy Reliability	Large range of power on step of 2 min 10 s sprint all out
Drouet et al. [43]	Tacx Flow Ergotrainer (Tacx BV, The Netherlands) and Computrainer Pro	Calibration rig	x		-	100–600	80–130	Full Step	Accuracy	Power range—Cadence range
	SRM and PowerTap	Calibration rig	x		-	330–607	80–130	Full Step		Power range—Cadence range
Duc et al. [77]	Ergomo Pro (G-Sensortrchnik GmbH und Co. KG, Mörfelden-Walldorf, Germany)	SRM	x	x	1	100–900	47–123	Peak and mean 1 and 5 min	Accuracy Reproducibility	Power range—Sprint—Cadence range
Earnest et al. [78]	Lode Excalibur (Lode, Groningen, The Netherlands)	-	x		12	100–300	Free	Last minute of each step	Reproducibility	Power range
Finn et al. [79]	Repco air-braked ergometer (Repco Ltd., Huntingdale, Victoria, Australia)	Calibration rig	x		-	150–1500	70–150	Full step	Accuracy Reproducibility (two ergometers and two trials) Sensitivity	Barometric pressure-Cadence range—Power range
Fiolo et al. [80]	Tire pressure sensor (Arofly X-Elite New Taipei City, Taiwan)	SRM	x		12	50–300	50–100	60 s (eliminating first and last 15 s of the original sample of 90 s)	Accuracy Reproductibility Sensitivity	Steady state riding on trainer with various gearing and cadence
Franklin et al. [81]	Monark 824E	SRM Professional	x		8	~180	60	Last 3.5 min averaged	Accuracy Repeatability	-
Gardner et al. [42]	SRM	Calibration rig	x		-	50–1000	60–120	Full step, averaged periods ≥1 min and peak power	Accuracy Reproducibility Robustness	Calibration—temperature—cadence—power range
	PowerTap	Calibration rig	x		-	50–1000	60–120	Full step, averaged periods ≥1 min and peak power		Calibration—temperature—cadence—power range
Glaner and Sliva [82]	ICBE (Home made)	Monark 874 Gaz exchange Metalyzer 3B (Cortex Byophisic)	x		42	50–500	50	Peak VO_2_, HR RPE	Validity	Step test (Balke protocol)
Gordon et al. [83]	Polar S710 (Polar, Kempele, Finland)	SRM	-		-	-	-	-	-	Mechanical issues related to the use of the Polar S710
Granier et al. [84]	Stages one-sided	SRM Professional	x		11	100–1200	Free during submaximal test ~120 rpm at the end of each sprint	Last 30 s of each step during submaximal test First 5 s of each sprint	Accuracy Reproducibility (two trials)	Power range—Sprint
Hoon et al. [85]	Wahoo KICKR (Wahoo Fitness, Atlanta, GA, USA) + SRM	Calibration rig	x		Calibration rig	50–400	Free	Final 10 s of each step	Accuracy Reproducibility (five trainers and two trials by trainer)	Power range
Guiraud et al. [45]	Ergomeca friction-loaded ergometer (GP400, La Bayette, France)	MOXUS Modular VO_2_ System II (AEI Technologies, PA)	x		5	100–300	75–90	Last minute of each step	Accuracy	Power range—Participant
	Lifecycle 9500HR lifefitness (electromagnetic brake, Schiller Park, IL, USA)	MOXUS Modular VO_2_ System II	x		5	100–300	75–90	Last minute of each step		Power range—Participant
	Monark 824E	MOXUS Modular VO_2_ System II	x		5	100–300	75–90	Last minute of each step		Power range—Participant
	Polar S710	MOXUS Modular VO_2_ System II	x		5	100–300	75–90	Last minute of each step		Power range—Participant
	Computrainer Pro RC1 model 8001	MOXUS Modular VO_2_ System II + Prony calibration device	x		5	100–300	75–90	Last minute of each step		Power range—Participant
Hopker et al. [86]	Wattbike Cycle Ergometer (Wattbike Ltd., Nottingham, UK)	SRM Scientific	x		Calibration rig	50–1250	70–90 up to 700 W 90 above 700 W	Last minute of each step	Accuracy Reproducibility	Power range—Cadence range
	Wattbike Cycle Ergometer	SRM Scientific			20	50–340	70–105	Full step		Participant-Power range—Cadence range
Hurst and Atkins [87]	Polar S710	SRM Amateur	x		12	~550	~115	Max and full step	Accuracy	Power range
Hurst et al. [88]	Stages one-sided	SRM Professional		x	1	~230–450	~75–102	Max and full step	Accuracy Reproducibility (15 trials)	Peak and mean power of one off-road climb
Randolph et al. [89]	Garmin Vector	SRM Scientific	x	x	1	100–1150	47–123	Max and full step	Accuracy Reproducibility (10 trials)	Power range—Cadence range—Torque range—Field—Position—Sprint
Jones and Passfield [90]	SRM (2 Scientific and 1 Professional)	Monark 824E ergometer	x		-	90–630	90	Last minute of each step	Accuracy Repeatability (two trials)	Power range
Kirkland et al. [91]	Ergomo pro	SRM + Monark 814E	x		9	50–450	Free (~80)	Full step	Accuracy Repeatability (three trials)	Patten range—Power range—Balance L/R quantified with Lode
Lawton et al. [92]	SRM	Calibration rig	x		19 SRM1 Cal rig	50–900	100	Unknown	Accuracy Reproducibility	Power range
Lanferdini et al. [93]	Garmin Vertor	Lode Excalibur Sport	x		14	100–400	95 ±5	60 s	Accuracy reliability	Rampe test steady cadence Power range
Lillo-Bevia and Pallares [94]	Hammer Cycle Ergometer (CycleOps, Madison, WI, USA)	SRM Scientific	x		11	100–500	70–100	Full step excepted first 10 s and last 5 s	Accuracy Reproducibility (three trainers)	Power range—Cadence range—Position
MacIntosh et al. [95]	Monark (834E and 868) ergometers	-	x		5	600–1200	Free	Peak and mean 30 s (Wingate)	Accuracy	Sprint (Wingate)
Maier et al. [96]	SRM (12) Powertap (10) Quarq (11) Stages (13) Verve (3) Power2max (2) Garmin Vector (1) Keo Power (1) RotorPower(1)	Mathematical model	x		32	Up to 360	Free	Full step	Accuracy Repeatability (three trials) Reproducibility between power meters	Power range
McGowan and Watson [97]	Wahoo KICKR Garmin, Pioneer, Quarq, SRM, Stages	Calibration rig	x		20	50–1000	90–120	Middle 30 s of each step Peak power for each sprint	Accuracy	Power range—Cadence range—Sprint—Time
McGregor and Rivera [98]	Computrainer	-	x		14	350–600	Free	Mean every 5 s and over the entire test	Accuracy Reproducibility (two trials)	Sprint (Wingate)
	Powertap	-	x		14	350–600	Free	Mean every 5 s and over the entire test		Sprint (Wingate)
Maxwell et al. [99]	35 friction-braked (Monark) ergometers	Calibration rig	x		Calibration rig	50–400	60	Last minute of each step	Accuracy Robustness	Power range—Oldness (old vs. new)—hysteresis effect (up vs. down)
	Five research-grade air-braked (Repco) ergometers	Calibration rig	x		Calibration rig	50–1150	50–150	Last minute of each step		Participant—Cadence range—hysteresis effect (up vs. down)
	Five electromagnetically braked ergometers	Calibration rig	x		Calibration rig	50–400	40–60	Last minute of each step		Participant—Cadence range
Merkes et al. [100]	Velocomp PowerPod (Velocomp LLC, Jupiter, FL, USA)	Verve Cycle InfoCrank (Verve Cycling, Perth, Australia)		x	12 and 4	100–1500	Free	Full step	Accuracy	Power range—Sprint
Micklewright et al. [101]	Lode Excalibur	Monark 814E	x		15	400–950	Free	Peak, minimum, and mean 30 s (Wingate)	Accuracy	Sprint (Wingate)
Miller et al. [102]	Quarq (Quarq, Spearfish, SD, USA) and Stages one-sided	PowerTap	x		4	~220	60–90	Full step	Accuracy	Participant—Cadence range
	Quarq and Stages one-sided	PowerTap		x	8	100–350	50–80	Full step according to the three profiles (UP, DH, and Flat)		Participant—Power range—Field profile-Frequency distribution
Millet et al. [103]	Polar S710	SRM Professional		x	8	200–440	Free	Max 5-s value and full step	Accuracy Repeatability (four trials) Sensitivity	Participant—Power range—Position
Polar S710	SRM Professional	x		44	~130–170	60–110	Last 5 min of each trial		Participant-Cadence range	
Nimmerichter et al. [104]	Garmin Vector	SRM Professional	x	x	6	100–850	50–110	Full step, 30-s intervals and peak 1 s	Accuracy Repeatability	Power range—cadence range—sprint-time
Montalvo-Pérez et al. [105]	Favero Duo	SRM			33	1000–1500 Sprint	75–100	By step of 75 s	Accuracy Reliability	Power range at different cadence and all out sprint test.
Novak et al. [40]	Lemond revolution cycle ergometer (HOIST Fitness, CA, USA)	SRM Scientific	x		10	100–400	Free	Full step	Accuracy	Participant—Power range
	Lemond revolution cycle ergometer	SRM Scientific	x		9	100–1400	Free	Peak 5 s (stationary and rolling) and 15 s seated + mean 5 s (stationary and rolling), 15, 30, 60, 240, and 600 s rolling		Participant—Power range—sprint with standing start (flywheel speed with both stationary and rolling starts)
Novak and Dascombe [37]	Garmin Vector	SRM Scientific	x		21	200–1200	Free	Peak 5 s (stationary and rolling) and 15 s seated + mean 5 s (stationary and rolling), 15, 30, 60, 240, and 600 s rolling	Accuracy	Participant—Power range—sprint with standing start (flywheel speed with both stationary and rolling starts)
Pallares and Lillo-Bevia [106]	PowerTap P1	SRM Scientific	x		33	100–500	70–100	Full step excepted first 10 s and last 5 s	Accuracy Reproducibility	Power range—Cadence range—Position
Paton and Hopkins [107]	Kingcycle Air-Braked Ergometer, SRM and PowerTap	-	x		11	Unknown	Free	Mean 5-min time-trial	Reproducibility (three trials)	-
Gross et al. [108]	PowerCal	Velotron Ergometer	x		9	100–300	~90	Full step	Accuracy Reproducibility (two trials)	Power range
Peiffer and Losco [109]	Tacx Fortius Virtual Reality cycle trainer	PowerTap	x		10	150–375	Free	Full step and 2 km averaged values during TTs	Accuracy Reproducibility (three TTs of 20 km)	Participant—Power range
Reiser et al. [110]	Cyclus (Avantronic, Leipzig, Germany) and Lode Excalibur	SRM	x		12	100–250	70–90	Last minute of each step-Indirect comparison with physiological measurements	Accuracy	Power range—Cadence range
Reiser and Hart [111]	Kreitler Alloy roller (Kreitler Custom Rollers, CO, USA)	SRM Professional	x		1	0–450	60–110	Last minute of each step	Accuracy	Power range
Rivera and McGregor [112]	Computrainer	-	x		14	200–250	Free	Full step	Accuracy Reproducibility (two trials)	Power range
	Powertap	-	x		14	200–250	Free	Full step		Power range
Rodger et al. [113]	Cyclus	SRM	x		10	140–1000	Free for incremental and sprint tests 100 for isokinetic test	Full step for incremental and isokinetic tests Mean 10 s for sprint test	Accuracy	Power range—Sprint
	Stages one-sided	SRM	x		10	140–1000	Free for incremental and sprint tests 100 for isokinetic test	Full step for incremental and isokinetic tests Mean 10 s for sprint test		Power range—Sprint
Rodrigez-Rielves et al. [114]	Favero DUO	SRM	x		12	100–650	70–100	Full Stage and peak value	Accuracy Reliability Robustness	Power range seating and standing position with vibration (20–40 Hz)
Rodrigez-Rielves et al. [115]	Rotor 2INpower	SRM	X		12	100–650 Spint >1000	70–100	Full Stage and peak value	Accuracy Reliability Robustness	Power range seating and standing position with vibration (20–40 Hz)
Sparks et al. [38]	Look Keo Power Pedals (Look, Cadex, France)	SRM Scientific	x		10	75–1100	80–90 Free during sprints	Final 30 s of each step of the incremental protocol Each 3 min, 100-W stage of the repeated-sprint protocol Mean of the first 5 s of all-out bouts	Accuracy Reproducibility (two trials for both incremental and sprint tests)	Power range—Sprint
Wainwright et al. [116]	10 Wattbikes	Lode Excalibur	x		Calibration rig	100–1000	70–130	Last 30 s of each step	Accuracy Reproducibility (10 Wattbikes and two trials by Wattbike)	Power range—Cadence range
Whittle et al. [117]	PowerTap P1	Wattbike Cycle Ergometer	x		10	0–600	Free	Full step Peak	Accuracy Reproducibility (three trials)	Power range—Time
Wilmore et al. [118]	Friction-braked ergometer (fabric belt)	-	x		10	50–250	60	Full step	Accuracy Reproducibility inter-ergometers	Power range
	Friction-braked ergometer (disc brake)	-	x		10	50–250	60	Full step		Power range
	Electrically-braked ergometer	-	x		10	50–250	60	Full step		Power range
	Friction-braked ergometer (hydraulic system)	-	x		10	50–250	60	Full step		Power range
Wright et al. [119]	Powertap P1	Lode Excalibur				100–250 W Sprint 10 s		2 min step Spirnt 10 s average	Accuracy Reliability	Power range and sprint comparison between brand new and after 100 h of use
Woods et al. [41]	Monark	Calibration rig	x		Calibration rig	0–350	60	Last minute of each step	Accuracy Repeatability (six trials) Reproducibility (six trials)	Power range
Zadow et al. [120]	Wahoo KICKR	Calibration Rig	x		Calibration Rig	100–1000	80–120	Last minute of each step	Accuracy	Power range—Cadence range
Zadow et al. [121]	Wahoo KICKR	Calibration Rig	x		Calibration Rig	100–600	80–100	Last minute of each step	Accuracy Robustness	Power range—Cadence range

**Table 2:** The various statistical methods used in validity studies regarding power meters are presented in Table 1.

Type of Analysis	Number of Studies
Hypothesis test for bias (Paired *t*-test, ANOVA)	54
Correlation coefficient and linear regression	32
Limits of agreement	46
Effect size	7
CV	33
ICC	13

ANOVA: Analysis of variance; CV: Coefficient of variation; ICC: Intraclass correlation coefficient.
